# Freezing Does Not Alter Sperm Telomere Length despite Increasing DNA Oxidation and Fragmentation

**DOI:** 10.3390/genes14051039

**Published:** 2023-05-03

**Authors:** Charlène Gouhier, Hanae Pons-Rejraji, Sandra Dollet, Laure Chaput, Céline Bourgne, Marc Berger, Bruno Pereira, Andrei Tchirkov, Florence Brugnon

**Affiliations:** 1 CHU Clermont-Ferrand, Laboratoire AMP-CECOS, F-63003 Clermont-Ferrand, France; cgouhier@chu-clermontferrand.fr (C.G.); lchaput1@chu-clermontferrand.fr (L.C.); fbrugnon@chu-clermontferrand.fr (F.B.); 2 UMR 1240 INSERM, IMoST, Université Clermont Auvergne, F-63000 Clermont-Ferrand, France; sandra.carlet_dollet@uca.fr; 3 CHU Clermont Ferrand, Laboratoire d’Hématologie Biologique, F-63003 Clermont-Ferrand, France; cbourgne@chu-clermontferrand.fr (C.B.); mberger@chu-clermontferrand.fr (M.B.); 4 CHU Clermont-Ferrand, DRCI-Délégation Recherche Clinique et Innovation, F-63000 Clermont-Ferrand, France; bpereira@chu-clermontferrand.fr; 5 CHU Clermont-Ferrand, Service de Cytogénétique Médicale, F-63003 Clermont-Ferrand, France; atchirkov@chu-clermontferrand.fr

**Keywords:** human spermatozoa, sperm telomere, Q-FISH, cryopreservation, nuclear alterations

## Abstract

Correlations were reported between sperm telomere length (STL) and male fertility, sperm DNA fragmentation, and oxidation. Sperm freezing is widely used for assisted reproductive techniques, fertility preservation, and sperm donation. However, its impact on STL remains unknown. For this study, semen surplus from patients who underwent routine semen analysis were used. The impact of slow freezing on STL was analyzed by performing qPCR before and after freezing. Sperm populations with different STL were evaluated using Q-FISH. The relationship between sperm DNA oxidation, DNA fragmentation, and STL was assessed in fresh and frozen sperm samples. No significant impact of slow freezing on STL was observed, neither measured by qPCR nor Q-FISH. However, Q-FISH allowed for the distinguishing of sperm populations with different STLs within individual sperm samples. Slow freezing induced different STL distributions for some of the analyzed sperm samples, but no correlation was found between STL and sperm DNA fragmentation or oxidation. Slow freezing does not alter STL despite increasing sperm DNA oxidation and fragmentation. As STL alterations could be transmitted to offspring, the lack of impact of the slow freezing method on STL ensures the safety of this procedure.

## 1. Introduction

Telomeres are repeated non-coding hexameric 5′-TTAGGG-3′ tandem DNA sequences located at the end of chromosomes with a single-stranded guanine-rich region [1] at the end. Telomeres ensure stability during chromosome meiotic segregation and protect from the degradation of gene-rich regions [2,3]. Human male germ cells maintain their telomeres by telomerase, which regenerates hexameric sequences at the chromosomal ends [4]. During spermatogenesis, the increase in telomere length is observed from the spermatogonia to spermatozoa stage [5]. Shorter STL is associated with male infertility [6,7,8], decreased fertilization rate [9], and embryo quality in in vitro fertilization [10]. Patients with oligozoospermia were shown to have shorter telomeres than normozoospermic patients [11,12]. Several studies showed that sperm telomere length (STL) can be considered as a marker of sperm quality related to male fertility potential, for recent reviews see [13,14]. Moreover, the association between STL and male fertility is supported by the correlation observed between STL and sperm DNA fragmentation [15,16,17] and sperm DNA oxidation [7,18,19]. Telomeres are sensitive to oxidative stress due to their particularly rich guanine structure. Reactive oxygen species (ROS) generate oxidized DNA-based adducts within the DNA strand, such as 8-hydroxy-2′-deoxyguanosine (8-OHdG). This results in a DNA strand break because spermatozoa contain only one base excision repair enzyme, 8-oxoguanine DNA glycosylase (OGG1), which excises the base adduct from the DNA sequence and may induce DNA fragmentation [20,21].

In previous studies, different techniques were used to measure STL. The Southern blot method was the reference technique until the development of quantitative polymerase chain reaction (qPCR), which is now the most often used technique because it is highly sensitive, easy to apply, and the fastest way of measuring the average relative telomere length of a whole sample [22]. Quantitative fluorescence in situ hybridization (Q-FISH) [23] permits spermatozoa populations to be differentiated according to their telomere lengths and to analyze individually spermatozoon telomere length.

Slow sperm freezing is widely used on a routine basis for assisted reproductive techniques (ARTs), fertility preservation, and sperm donation [24]. It is well established that this technique leads to sperm damage due to oxidation [25,26] and may induce a decrease in STL. Therefore, it is possible that freezing affects STL by inducing oxidative stress. The objective of this study was to measure the impact of freezing human sperm on STL measured using qPCR and Q-FISH techniques. In parallel, we measured sperm DNA fragmentation and oxidation to identify possible relationships with STL during sperm freezing.

## 2. Materials and Methods

### 2.1. Ethical Approval

Written informed consent was obtained for inclusion of the semen samples from patients in the Germetheque biobank with the approval of the local committee (Committee for Personal Protection DC 2008 558 number: 20200703, Trial registration number: NCT04715828) on 6 August 2020. The study was conducted in accordance with the principles of the Declaration of Helsinki.

### 2.2. Experimental Design

Thirty patients who underwent routine semen analysis for infertility at the Center for Reproductive Medicine of the University Hospital of Clermont-Ferrand were recruited. Only men with no significant concentration of polymorphonuclear leukocytes (PMN), (under 5% of spermatozoa concentration), were included. After semen analysis using the fresh sample, the semen samples were frozen with slow freezing. The samples were stored in the Germetheque biobank. Standard semen parameters [27] and STL measured by qPCR were analyzed for the 30 fresh and frozen sperm samples. To analyze the remaining markers (STL by Q-FISH; DNA fragmentation by TUNEL; DNA oxidation by immunodetection of 8-OHdG residues), before and after freezing, we selected 10 samples with a high number of spermatozoa (70.10^6^ spermatozoa per ejaculate).

### 2.3. Standard Semen Analysis

Standard semen analysis was performed according to WHO guidelines [27] after 2–7 days of sexual abstinence. PMN were detected using LeucoScreen kits (Fertipro, Beernem, Belgium). Sperm vitality was measured using VitalScreen^®^ (Fertipro, Beernem, Belgium). Morphology was evaluated in fresh samples according to Kruger’s classification [28]. Oligozoospermia (sperm concentration under 15.10^6^ spermatozoa per mL or sperm number lower than 39 × 10^6^ per ejaculate), necrozoospermia (vitality under 58%), asthenozoospermia (total motility under 40% or progressive motility under 32%), teratozoospermia (percentage of spermatozoa with typical morphology under 4%), and leukocytospermia (concentration of PMN higher than or equal to 1 × 10^6^ per mL) were defined according to WHO guidelines [27].

### 2.4. Sperm Freezing and Thawing

Samples were mixed in a 1:1 ratio (*v*/*v*) with a cryoprotective medium (Cryosperm, Origio, Malov, Denmark) according to the supplier’s recommendations. Afterwards, sperm samples were frozen in high security straws (Cryobiosystem^®,^ L’Aigle, France) in a NanoDigitcool^®^ programmable freezer (Cryobiosystem^®^) and stored in a liquid nitrogen tank at −196 °C (Cryodiffusion^®^, Lery, France) until use (minimum few hours and maximum one month). The straws were thawed at 37 °C for 4 min. After a multi-step addition of an equal volume of Sperm Preparation Medium^®^ (Origio Malov, Danemark), the cryoprotectant was removed by a first wash by centrifugation (750× *g*, 5 min, RT). It was followed a second wash with 1 mL of Sperm Preparation Medium^®^ (Origio Malov, Danemark, 750 g, 5 min, RT).

### 2.5. Sperm Telomere Length Measurements

#### STL Measurement by qPCR

Sperm cells were washed by centrifugation (700× *g*, 8 °C, 7 min) in 1× phosphate-buffered saline (PBS; Thermo Fisher Scientific, Scotland, UK) and stored at −20 °C until DNA extraction for qPCR analysis. Genomic DNA extraction was performed using the Maxwell^®^ 16 Blood DNA Purification Kit, (Promega, Charbonnières-les-Bains, France). DNA samples (10 ng) were analyzed by qPCR on a LightCycler to determine the telomere repeat copy number (T) and the single copy gene copy number (S). The GAPDH gene was used as the single-copy reference gene. Two mixes containing Mastermix (LightCycler^®^ 480 SYBR Green I Master, Roche Diagnostics, Germany) and primers were produced, one with GAPDH primers and the second with the telomeric TELFR primers. The sequences of telomere-specific primers were as follows: Tel-F, CGGTTTGTTTGGGTTTGGGTTTGGGTTTGGGTTTGGGTT and Tel-R, GGCTTGCCTTACCCTTACCCTTACCCTTACCCTTACCCT. The GAPDH gene was amplified using the following primers: GAPDH-F, CCCCACACACATGCACTTACC and GAPDH-R, CCTAGTCCCAGGGCTTTGATT. PCR was performed in duplicate for GAPDH and triplicate for telomeric DNA. Negative (H2O) and positive (normal DNA in blood leukocytes) controls and standards with known copy numbers for GAPDH, and telomeric copy repeats were included in each PCR series. The absolute telomere repeats and reference gene copy numbers were calculated using standard dilution curves. The T/S ratio was then calculated and normalized using the normal control DNA. Samples were analyzed at different time points.

### 2.6. STL Measurement by Q-FISH

Sperm cells were fixed with methanol/acetic acid (3:1), spread on slides, and dried in a Thermotron. The slides were then treated with pepsin and washed in PBS (2 min), dehydrated in ethanol (70%, 90%, and 100% successively, for 5 min each), and air dried. The hybridization solution included 0.2 µM of C-rich telomere peptide nucleic acid (PNA) probe labeled with cyanine 3 (Eurogentec, Seraing, Belgium), 0.02 μM of centromere PNA probe labeled with FAM (Eurogentec, Belgium), deionized formamide ≥99.5% (Sigma-Aldrich, Saint Quentin Fallavier, France), a 1M solution of Trizma Hydrochloride^®^ (Sigma-Aldrich, Saint Quentin Fallavier, France) at pH 7.2, and blocking reagent. The slides were then heated (80 °C, 3 min) for the denaturation step and incubated in the dark at 37 °C. Next, the slides were treated with non-deionized formamide (≥99%, Honeywell^®^, Fisher Scientific, Illkirch-Graffenstaden, France), 5% BSA, and 10 mM Trizma Hydrochloride^®^ solution (Sigma-Aldrich, Saint Quentin Fallavier, France). Afterwards, the slides were rinsed in a solution of 0.08% Tris and dehydrated. Nuclei staining was performed using DAPI (DiAmino Phénol Indol, Fisher Scientific, Illkirch-Graffenstaden, France). The slides were analyzed using a fluorescence microscope (Axioplan2 imaging fluorescence microscope ZEISS, Göttingen, Germany) with specific fluorochrome filters. Cyanine3 and FAM fluorochromes were revealed using tetramethylrhodamine (TRITC) and fluorescein isothiocyanate (FITC) filters. In-depth images were obtained using a CCD camera and an Isis digital imaging system, v3.8.8 (Metasystems, Althussheim, Germany). The same software was used to measure the fluorescence intensity. The telomere to centromere ratio (TCR; i.e., the fluorescence values revealed by TRITC [red] and FITC [green]) was calculated for each nucleus. The TCR was obtained for 100 nuclei per slide.

### 2.7. TUNEL Assay

PBS-washed sperm cells were fixed with 4% paraformaldehyde and stored at 4 °C until treatment for the DNA fragmentation measurement. Sperm DNA fragmentation was measured using the TUNEL detection assay (Cell Death Detection Kit POD^®^, Roche, France) as reported previously [29]. Briefly, aliquots of fixed sperm cells were washed in PBS, followed by permeabilization in 100 µL of a solution containing 0.1% Triton X-100 in 0.1% sodium citrate for 2 min on ice. Labeling was performed after washing with PBS containing 1% BSA (1000 g, 5 min). Counterstaining with propidium iodide (PI) allowed evaluation of sperm permeabilization. Negative controls were obtained by incubating sperm cells without enzyme (terminal deosynucleotidyl transferase [TdT]). Positive controls were performed by incubating sperm cells with Dnase I enzyme (3 UI, Roche, France). Analysis was performed on a minimum of 20,000 cells using a BD FACS Aria SORP cytometer (BD Biosciences, Franklin Lakes, NJ, USA).

### 2.8. 8-OHdG Immuno-Detection

DNA oxidation was measured by 8-OHdG residue immuno-detection as described previously by [30]. Briefly, after decondensation with 2 mM dithiothreitol (DTT), 0.5% Triton X-100, and PBS, samples were fixed in 4% paraformaldehyde. Sperm samples were incubated with 1.5% normal goat serum solution before incubating with mouse anti-8-OHdG monoclonal primary antibody (Novus Biological^®^, Nantes, France) overnight at 4 °C. The sample was then incubated with secondary antibody IgG (P.A.R.I.S anticorps, Paris, France) coupled with Alexa 488 (Molecular Probes^®^, Eugene, OR, USA) for 45 min. For each assay, negative controls were obtained by incubating sperm cells without anti-8-OHdG primary antibody. Positive controls were performed by treating sperm samples with 8M H2O2 solution before fixation. The samples were analyzed on a minimum of 20,000 cells using a BD FACS Aria SORP cytometer (BD Biosciences, Franklin Lakes, NJ, USA). The proportion of sperm containing 8-OHdG and the mean fluorescence intensity (MFI) of oxidation were measured.

### 2.9. Statistical Analysis

Sample size was estimated according to Cohen’s recommendations, which define effect size bounds [31]. Indeed, in order to highlight an effect size greater than 1, at least 10 patients were necessary for each technique (qPCR and Q-FISH), and with an intra individual correlation coefficient at 0.50, a two-sided type I error equals 5% and 80% statistical power. For qPCR, 30 sperm samples were used in order to show an effect size around 0.5 with aforementioned assumptions for type I error, statistical power, and intra individual correlation coefficient. The continuous variables are presented as mean and standard error of the mean (SEM). To evaluate the effect of the initial STL value, we discriminate for qPCR and Q-FISH analyses samples having initial small STL and samples having higher STL (using the median value). The assumption of normality was assessed by the Shapiro–Wilk test. The categorical variables are presented as the number of patients and percentages. For paired comparisons, a (paired) Student t-test or Wilcoxon test was used. When analyses were conducted with several measures for a same subject (for Q-FISH in fresh and frozen), mixed models were used to take into account between and within subject variability (as random effect). The normality of residuals was analyzed as aforementioned. For all analyses, the results are expressed using effect sizes (ESs) and 95% confidence intervals (IC), and were interpreted according to Cohen’s recommendations, which define ES bounds as small (ES: 0.2), medium (ES: 0.5), and large (ES: 0.8, “grossly perceptible and therefore large”). The relationships between quantitative variables were analyzed by correlation coefficients (Pearson or Spearman according to the statistical distribution), applying Sidak’s type I error correction. The results were interpreted according to the following rules of thumb [32]: <0.3 is negligible correlation, 0.3–6 is low to moderate correlation, and >0.6 is moderate to high correlation. All analyses were performed in Stata software (version 15, StataCorp, College Station, TX, USA) for a two-sided type I error of 5%. As analyses were exploratory, the individual *p*-values are reported without systematically applying mathematical correction [33], but with specific attention paid to the magnitude of differences and the clinical relevance.

## 3. Results

### 3.1. Patient Characteristics, Sperm Analysis, and STL

Table 1 shows the clinical characteristics and semen parameters of the 30 men whose samples were evaluated in this study. Semen volume, spermatozoa concentration, progressive motility, and vitality were normal according to the WHO’s criteria, except one patient with necro-asthenozoospermia (progressive motility 3.5% and vitality 10%) and another patient with oligozoospermia (concentration 11.9 M/mL). The percentage of typical spermatozoa forms was normal for 19 patients. We found a mean STL measured by qPCR of 3.2 ± 0.2 arbitrary units (a.u., Table 1), with no significant difference between patients with altered sperm parameters compared to normozoospermic patients (3.5 ± 0.3 vs. 2.9 ± 0.3 a.u.

**Figure 1:**
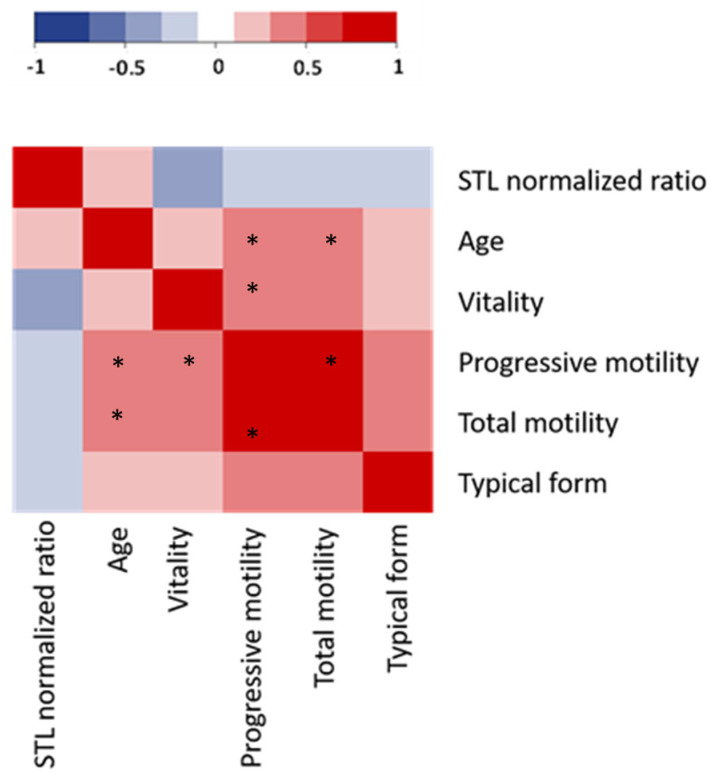
Heatmap of the correlation between STL measured by qPCR (STL normalized ratio) and spermatic and clinical characteristics for fresh samples. Spearman correlations: negative correlations appear in blue and positive correlations in red. No significant correlation was observed between STL and clinical and semen parameters; * indicates significant correlations.

We did not observe any correlation between STL and clinical characteristics, nor between STL and the measured semen parameters (Figure 1).

### 3.2. Impact of Freezing on Sperm Parameters and STL

After thawing, we observed a significant decrease in sperm progressive motility (12.6% vs. 48.5%, *p* < 0.001, Table 2 vs. Table 1) and vitality (37.8% vs. 73.1%, *p* < 0.001), with respective ESs of -2.8 [-3.8; -2.0] and -1.9 [-2.6; -1.3]. The mean STL was not different in fresh sperm and thawed samples (3.2 ± 0.2 vs. 3.3 ± 0.2 a.u.) when STL was measured by qPCR (n = 30). Secondly, we discriminated samples having the smaller STL mean before freezing (2.3 ± 0.1 a.u., n = 13) and samples with longer STL (3.9 ± 0.2 a.u., n = 17) in order to measure a potential effect linked to initial telomere length. The mean STL was not significantly different in fresh and thawed samples for the two groups (2.4 ± 0.2 for smaller STL group and 3.9 ± 0.2 for longer STL group).

**Figure 2:**
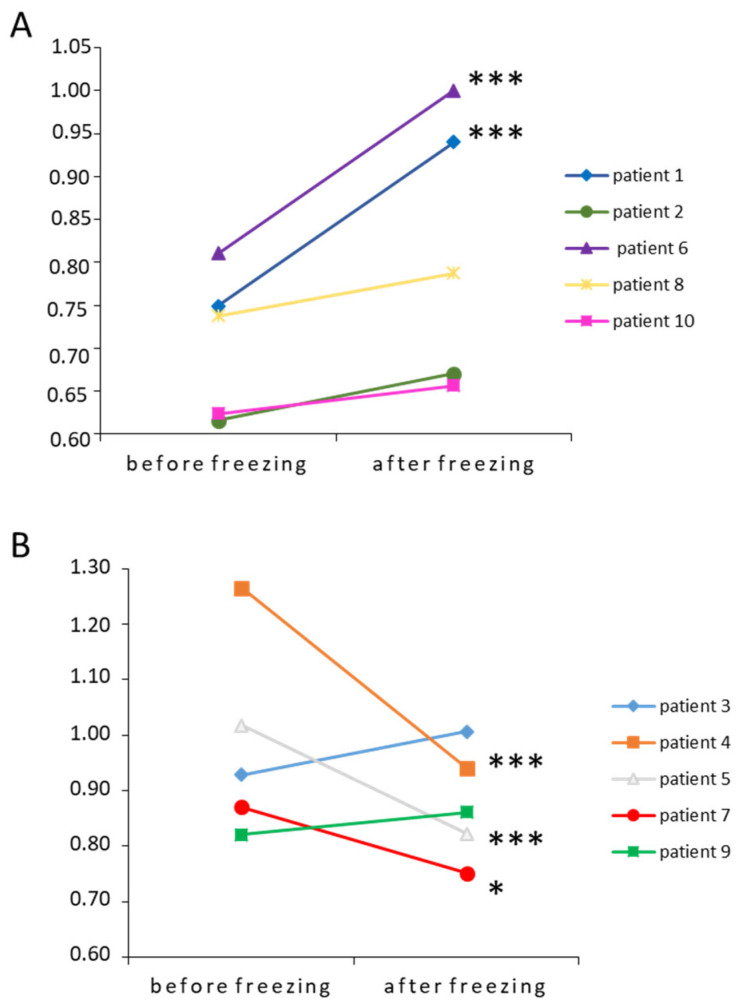
Individual impact of freezing-thawing cycle on samples with the smaller (**A**) and longer (**B**) STL measured by Q-FISH. * and *** correspond to *p* < 0.05 and < 0.001, respectively, compared to fresh sperm samples.

Among the 30 sperm samples, STL was also measured by Q-FISH in 10 samples. There was no difference in STL between fresh and frozen sperm samples evaluated by Q-FISH (0.85 ± 0.06 a.u. vs. 0.85 ± 0.04 a.u.). As for qPCR analysis, we subdivided sperm samples in two groups: one with the samples having the shorter STL in mean and one with the longer STL. We measured no significant impact of freezing on the “shorter STL” group (0.72 ± 0.04 vs. 0.82 ± 0.07) and on the “longer STL” group (0.97 ± 0.08 before vs. 0.87 ± 0.04 after freezing). We also measured the individual impact on each sperm sample. Patients 1 and 6 showed significant increase in their STL (patient 1: 0.75 ± 0.02 vs. 0. 94 ± 0.05 and patient 6: 0.85 ± 0.3 vs. 1.0 ± 0.03 for, *p* < 0.001, Figure 2A). For both patients, we measured a strong decrease in vitality after freezing (patient 1: 76 vs. 43% and patient 6: 80 vs. 56%). Among the longer STL group, three patients have significant decreased values of STL after freezing (Figure 2B): patient 4 (1.26 ± 0.06 vs. 0.95 ± 0.04, *p* < 0.001), patient 5 (1.02 ± 0.04 vs. 0.83 ± 0.04, *p* < 0.001), and patient 7 (0.84 ± 0.03 vs. 0.75 ± 0.03, *p* < 0.05). The three patients presented a relatively low decrease in vitality after freezing (patient 4: 78 vs. 61%, patient 5: 76 vs. 62%, and patient 7: 86 vs. 70%).

**Figure 3:**
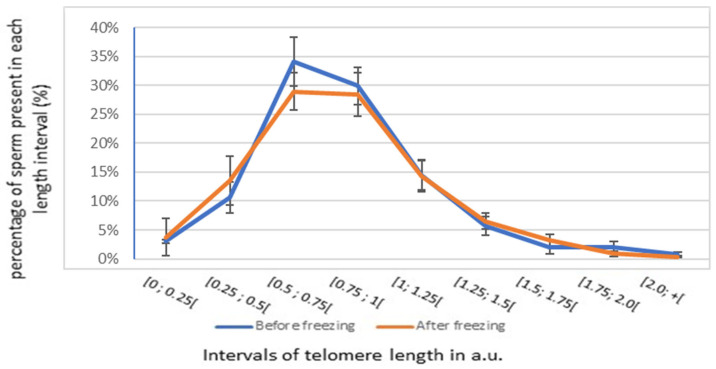
Mean STL distribution measured by Q-FISH before and after freezing–thawing. Mean distribution ± SD of the sperm proportion in each telomere length interval measured by Q-FISH before (blue) and after freezing (orange) in 10 patients.

Secondly, we segmented STL into intervals of 0.25 a.u. to quantify the proportion of spermatozoa present in each length interval. The results obtained with Q-FISH in fresh and frozen sperm samples are shown in Figure 3 (blue and orange, respectively). On average, most spermatozoa in fresh samples (64%) had a relative telomere length of 0.5 to 1 a.u. After thawing, 57% had a length of 0.5 to 1 a.u. We did not find a significant difference in the mean distribution of telomere length between fresh and frozen sperm samples (Figure 3).

We focused on the behaviors of sperm cells with the smaller STL in the 10 samples (corresponding to the percentage of sperm cells in the two first intervals of STL: [0; 0.25[ and [0.25; 0.5[). We measured no significant difference in the percentage of sperm cells having the smaller STL before (10.9 ± 2.7%, supplemental Figure S1),) and after cryopreservation (17.2 ± 7.1%), even if it tended to be higher after freezing. We observed a negative correlation between the proportion of spermatozoa with shorter STLs (corresponding to STL intervals: [0; 0.5[) after freezing and progressive (r = −0.88, *p* < 0.001, supplemental Figure S2) and total (r = -0.80, *p* < 0.05) motility measured after freezing. We also found a positive correlation between the proportion of sperm cells with longer STL (corresponding of intervals of STL [1; +[) after freezing and progressive (r = 0.82, *p* < 0.01, supplemental Figure S2) and total motility (r = 0.75, *p* < 0.05) measured also after freezing. For this second population ([1; +[), we observed a positive correlation between the proportion of sperm cells with long STL before and after freezing (r = 0.72, *p* < 0.05).

**Figure 4:**
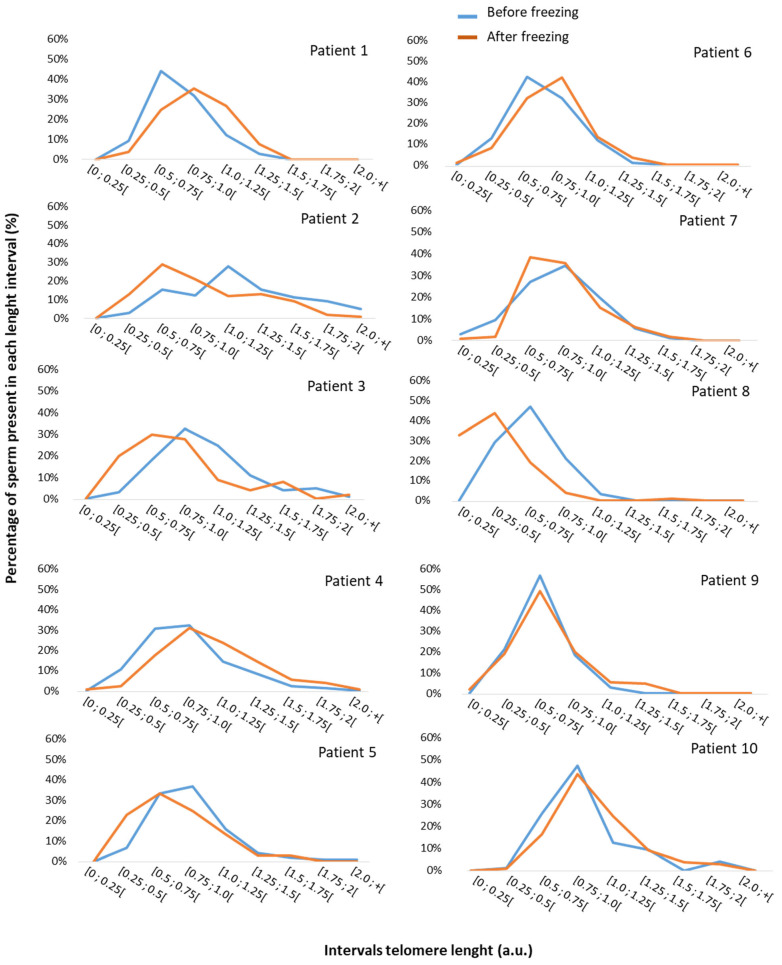
Individual STL distribution measured by Q-FISH before and after freezing–thawing. Individual sperm proportion in each telomere length interval before freezing (blue) and after freezing (orange). We observed that a larger proportion of spermatozoa had short STLs after freezing in three patients (patients 2,3 and 8).

We observed a large inter-patient variability both in the distribution of STL and in the impact of the freezing–thawing cycle (Figure 4). Indeed, by comparing the distribution of STL for each patient before and after freezing, we observed the presence of a larger proportion of spermatozoa with a short STL after thawing for three patients (patients 2, 3, and 8, Figure 4). In fresh samples, most of the spermatozoa from patient 2 had telomere lengths that varied from 1 to 1.5 a.u. (43%, Figure 4). After thawing, only 25% of the spermatozoa fell into this length category, whereas half (50%) had a length varying from 0.5 to 1 a.u. Regarding patient 3, 58% of the spermatozoa in the fresh sample had a telomere length ranging from 0.75 to 1.25 a.u. After thawing, only 37% of spermatozoa had this telomere length. Half of the thawed spermatozoa presented a length varying from 0.25 to 0.75 a.u., whereas only 21% of the fresh spermatozoa fell into this category (Figure 4). Patient 8 had spermatozoa with shorter telomeres than the average profile. In fresh samples, 47% of the spermatozoa had a telomere length varying from 0.5 to 0.75 a.u., and 29% of the spermatozoa were between 0.25 and 0.5 a.u. After thawing, 77% of the spermatozoa had a telomere length varying from 0.0 to 0.5 a.u. (Figure 4). The pattern observed for these three patients was not related to age, BMI, tobacco consumption, or the deterioration of a sperm parameter (*p* > 0.05). For patient 2, the standard sperm parameters were normal in fresh and thawed samples. For patient 3, we measured a lower progressive sperm motility after thawing compared to the fresh sample (62.5% vs. 18%) and normal vitality in the fresh and thawed samples (76% vs. 62%). For patient 8, while a decrease in sperm vitality (70% vs. 50%) was observed between fresh and thawed samples, normal motility was quantified in both samples (44% vs. 34%).

### 3.3. Relationship between Sperm DNA Oxidation, DNA Fragmentation, and STL in Frozen Sperm Samples

**Figure 5:**
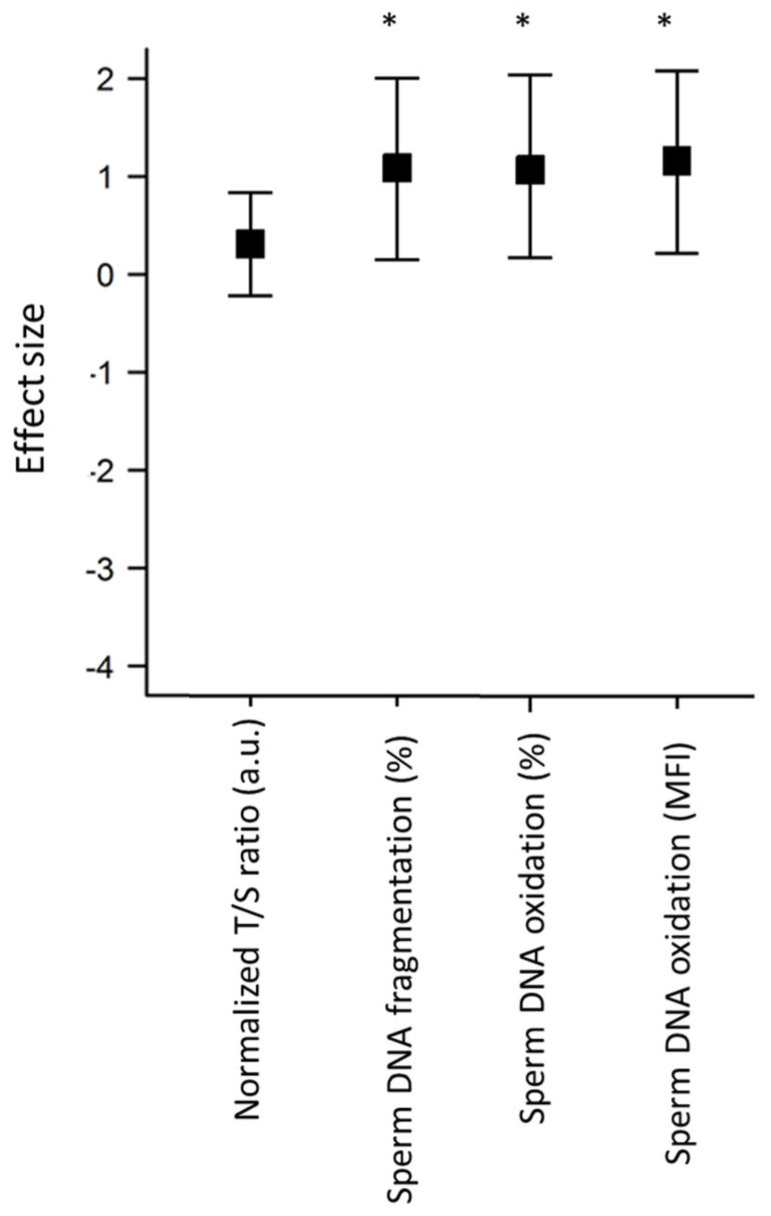
Impact of freezing on sperm telomere length (STL), DNA fragmentation, and oxidation. Forest plot representing the effect size with 95% confidence intervals of slow freezing–thawing on STL (n = 30) measured by qPCR, a well as on sperm DNA oxidation and DNA fragmentation (n = 10). MFI: mean fluorescence intensity. * *p* < 0.05 compared to fresh sperm samples. The error bars indicate confidence intervals.

We measured a higher proportion of spermatozoa with sperm DNA fragmentation (38.8 vs. 23.8, *p* < 0.05 Table 3), nuclear oxidation (85.1% vs. 73.2%, *p* < 0.05), and with MFI (1445.2 ± 202.1 vs. 839 ± 210, *p* < 0.05) in thawed samples compared to fresh samples, with respective ESs of 0.8 [−0.05; 1.8], 0.8 [−0.07; 1.7], and 0.9 [−0.01; 1.8] (Figure 5). No correlation was found between the STL measured by qPCR and the rate of sperm DNA fragmentation (r = 0.1, n = 10) or the percentage of oxidation (r = 0.18, n = 10). We did not measure any significant correlation between the different nuclear markers.

## 4. Discussion

To the best of our knowledge, this is the first study analyzing the impact of freezing on STL. Furthermore, we used two validated methods to analyze sperm DNA fragmentation and oxidation to better characterize the relationship between STL and sperm quality. The results of our study indicate that sperm freezing does not alter STL despite increasing sperm DNA oxidation and fragmentation. A high proportion of spermatozoa with short telomeres is associated with infertility, reduced fertilization, and poor embryo development [13,14]. Telomeres contribute to the early event of male pronuclear formation after oocyte activation since they are attached to the nuclear envelope [34]. Shortened telomeres would impair attachment of chromosomes to the nuclear envelope, which may induce a higher rate of aneuploidy and failure of pronuclear formation [35]. Moreover, telomere length is considered a heritable trait, though the modality of parental inheritance is not still completely understood [36]. As sperm freezing is used for fertility preservation [37], ART [24], and sperm donation [38], the lack of impact of freezing on STL seems to guarantee the safety of this technique. The results of our study strengthen the use of frozen/thawed sperm for ART. Nevertheless, when we measured STL by Q-FISH, we observed a different distribution of sperm populations with shortened telomeres in three patients without alterations in the standard semen parameters. This underlines the heterogeneity of telomere sensitivity in the different spermatozoa populations in an ejaculate. There is no significant effect of freezing on sperm samples with the shorter STL, either measured by qPCR or by Q-FISH. However, the proportion of spermatozoa with short STL ([0; 0.25[ and [0.25; 0.5[) tended to be higher after freezing. Two patients (1 and 6) showed a significant increase in STL measured by Q-FISH, which could be explained by the high impact of freezing on sperm vitality. Indeed, it is known that Q-FISH cannot measure telomere length of chromosomes in senescent or aberrant cells [39]. Since cell selection is based on fluorescence intensity, a bias targeting living cells can be supposed, inducing a non-specific increase in the average estimated STL value for samples having a low vitality.

The Q-FISH technique allows telomere length to be measured at the level of the individual telomere. This method is also highly sensitive for determining telomere length in samples limited to a small number of cells. Terminal restrictive fragment (TRF) based on Southern blot is considered the gold standard for measuring the absolute value of the telomeres within a DNA sample. Although this method is robust, highly repeatable, and reproductive, it is expensive and has limitations, requiring large amounts of DNA for the analysis and overestimating telomere length due to the inclusion of subtelomeric regions in the total length. For these reasons, different methods, such as Q-FISH and qPCR, were developed to determine relative telomere length. Quantitative PCR measures relative, rather than absolute telomere length, by generating a ratio of the total telomere DNA and DNA from the amplification of a single copy gene using the SYBR-green PCR technology. This method is quick, easy to apply, and requires a lower amount of DNA than TRF. The choice of technique to apply depends on the sample type and DNA amount. Quantitative PCR has the limitation of giving the STL average of a whole sperm sample without distinguishing sperm cells from other cell types. In our study, we ensured that the selected samples contained few PMN. Still, it is one limit of this analysis to not allow measurement of samples contaminated with PMNs. Moreover, studies showed that the extraction technique for qPCR can alter the telomere size results [40,41]. Indeed, as with all techniques, it is essential to carry out method validation, particularly in an analysis, for diagnostic purposes with internal controls. Q-FISH has the advantage of differentiating sperm from the other cells. It allows also for the highlighting of subpopulations within the same sample, which is not possible with qPCR, and thus it is possible to obtain better representation of STL heterogeneity. It gives strong and specific signals. However, it is a time-consuming technique. Chromosomes are isolated and hybridized with dye-labeled peptide nucleic acid probes, and fluorescence is quantified after manual isolation of each nucleus, giving a quantitative measure. This measure is normalized with a centromeres fluorescent signal and given finally a relative value (telomere/centromere signals ratio), rather than an absolute telomere length. For both techniques, an absolute quantification can be obtained by comparing STL to referent cells with known telomere length [39]. Q-FISH only assesses a small number of sperm nucleus (a few hundred) comparative to qPCR. A final limit of Q-FISH is that it cannot measure the telomere length of all chromosomes in terminally senescent or aberrant cells [39]. In conclusion, Q-FISH should be preferred for samples having a low number of spermatozoa, contamination with other cells, or when we need to characterize subpopulations. In the others cases, and notably for screening in routine analysis, qPCR is the fastest way of measuring average relative STL.

Several previous studies clearly showed that sperm freezing induces DNA fragmentation and oxidation [25,26,42]. With the guanine-rich regions in telomere DNA, it is more susceptible to oxidative stress, which is known to cause telomere shortening due to the oxidation of guanine bases to 8-OHdG and DNA fragmentation [6,20,21]. In our study, we observed a significant increase in sperm DNA oxidation and fragmentation without a decrease in STL after sperm freezing. These deleterious effects of sperm freezing due to oxidative stress are in agreement with previous results [25,26,43]. The absence of effects on STL suggests that oxidative DNA damage at telomeres can be efficiently protected in normal sperm. In particular, shelterin proteins protecting telomeres [3] enhance the base excision repair involved in the processing of oxidative DNA lesions [44]. Telomerase can also restore telomere losses induced by oxidative damage in somatic cells [45].

A limitation of our study is the large number of spermatozoa required to perform this multiparametric study. We selected sperm samples with high sperm concentrations in order to have sufficient spermatozoa to perform the analysis. Therefore, the majority of the sperm samples we analyzed had normal or subnormal standard semen parameters. This may explain why we did not observe any correlation between sperm DNA oxidation, fragmentation, and STL. It would be interesting to analyze whether sperm freezing has deleterious effects on STL in a comparative study of a large number of sperm samples from patients with oligo-astheno-teratozoospermia, which is more sensitive to oxidative stress [46]. This may provide more information on why we did not observe any correlation between these markers as reported by other studies in fresh sperm samples [8,17,19] and not confirmed by other authors [6,7,47]. For infertile couples, notably with men presenting altered sperm parameters, or following fertilization and pregnancy rates in IVF, default of early embryo development, and repeated miscarriage, STL measure can be a good candidate to explain potential idiopathic infertility or/and a marker to prevent a potential trans generational effect [13,14]. Moreover, it was recently demonstrated that STL is influence by lifestyle, diet, and sport activity [48], so it can be a global marker of good health that impacts pregnancy outcomes.

Finally, we did not observe any correlation between patient age and STL. This is in agreement with previous reports of patients of a similar age range [9,16,18,49]. Other studies highlighted a positive correlation between age and STL, but the selected male populations had a larger age difference [23,50]. Moreover, due to the high variation in TL between individuals, a significant correlation can be observed when evaluating a much higher number of samples. We did not observe a correlation between STL measured by qPCR and standard sperm parameters. Studies performed on similar patients as our cohort confirmed our results [6,49]. Only the studies of sperm samples with reduced standard semen parameters reported an important decrease in STL [8,12,17]. In the same way, in the present study, we measured a significant negative correlation between the proportion of cells having short STL after freezing and mobility of frozen spermatozoa.

In conclusion, the results of our study clearly show that sperm slow freezing increases DNA oxidation and fragmentation, but does not alter STL. Furthermore, the measurement of STL by Q-FISH enabled the distinguishing of spermatozoa populations with different STL within the same sperm sample. As potential sperm telomere alterations could be transmitted to offspring, the lack of impact of the slow freezing method on sperm telomere length ensures the safety of this routinely used procedure. Due to the multiparametric analysis we performed, we mostly studied sperm samples with normal sperm concentration. It would be interesting in the future to study the impact of slow freezing on STL in sperm samples from patients with oligoastheno-teratozoospermia to better evaluate the potential effect of oxidative stress.

## Figures and Tables

**Table 1:** Clinical characteristics and semen parameters.

Parameters	Mean ± SEM
Age (years, n = 30)	32.8 ± 1
BMI (kg/m^2^, n = 21)	27 ± 1
Sperm volume (mL, n = 30)	4.7 ± 0.4
Sperm concentration (M/mL, n = 30)	108 ± 14.2
PMN concentration (M/mL, n = 30)	0.12 ± 0.06
Progressive motility (%, n = 30) Total motility (%, n = 30)	48.5 ± 2.4 54.8 ± 2.5
Sperm vitality (%, n = 30)	73.1 ± 2.7
Typical forms (%, n = 30)	6.7 ± 1
STL (a.u., n = 30)	3.2 ± 0.2

%: percentage; SEM: standard error of the mean, M: million, and PMN: polymorphonuclear leukocytes.

**Table 2:** Impact of freezing–thawing on sperm parameters and STL.

Parameters	After Freezing-Thawing (Mean ± SEM)	Effect-Size [95% CI]
Progressive motility (%, n = 30)	12.6 ± 2.4	−2.8 [−3.5; −2.0]
Total motility (%, n = 30)	19.9 ± 2.4	−2.6 [−3.2; −1.9]
Sperm vitality (%, n = 30)	37.8 ± 3.8	−1.9 [−2.6; −1.3]
STL (a.u., n = 30)	3.3 ± 0.2	0.08 [−0.4; 0.6]

%: percentage; SEM: standard error of the mean, STL: sperm telomere length; a.u: arbitrary units; and CI: confidence interval.

**Table 3:** Clinical characteristics, semen parameters, sperm DNA fragmentation, and oxidation.

Parameters	Fresh (Mean ± SEM)	After Freezing-Thawing (Mean ± SEM)	Effect-Size [CI]
Sperm DNA fragmentation (%, n = 10)	23.8 ± 5.3	38.8 ± 5.7 *	0.8 [−0.05; 1.8]
Sperm DNA oxidation (%, n = 10)	73.2 ± 4.7	85.1 ± 3.7 *	0.8 [−0.07; 1.7]
Sperm DNA oxidation (MFI, a.u., n = 10)	839.2 ± 210.6	1445.8 ± 202.1 *	0.9 [−0.01; 1.8]

%: percentage; SEM: standard error of the mean; MFI: mean fluorescence intensity; a.u: arbitrary units; and CI: confidence interval. * *p* < 0.05 compared to fresh sperm samples.

## Data Availability

The data presented in this study are available on request from the corresponding author.

## Supplementary Materials

The following are available online at https://www.mdpi.com/article/10.3390/genes14051039/s1, Figure S1: Mean and individual STL distribution of the shortest STL [0; 0.5 a.u.[) measured by Q-FISH before and after freezing-thawing; Figure S2: Heatmap of the correlations between the proportion of sperm cells having the shorter [0; 0.5 a.u.[) and the longer STL [1; +[ measured by Q-FISH before and after freezing, and sperm parameters for frozen samples.
