# Supplementary material for: Freezing Does Not Alter Sperm Telomere Length despite Increasing DNA Oxidation and Fragmentation
Source: Genes (Basel). 2023 May 3;14(5):1039. doi: 10.3390/genes14051039 (PMC10218602; doi:10.3390/genes14051039)
Supplement: Supplementary file 1 [file genes-14-01039-s001.zip › genes-2305195-supplementary.pdf]

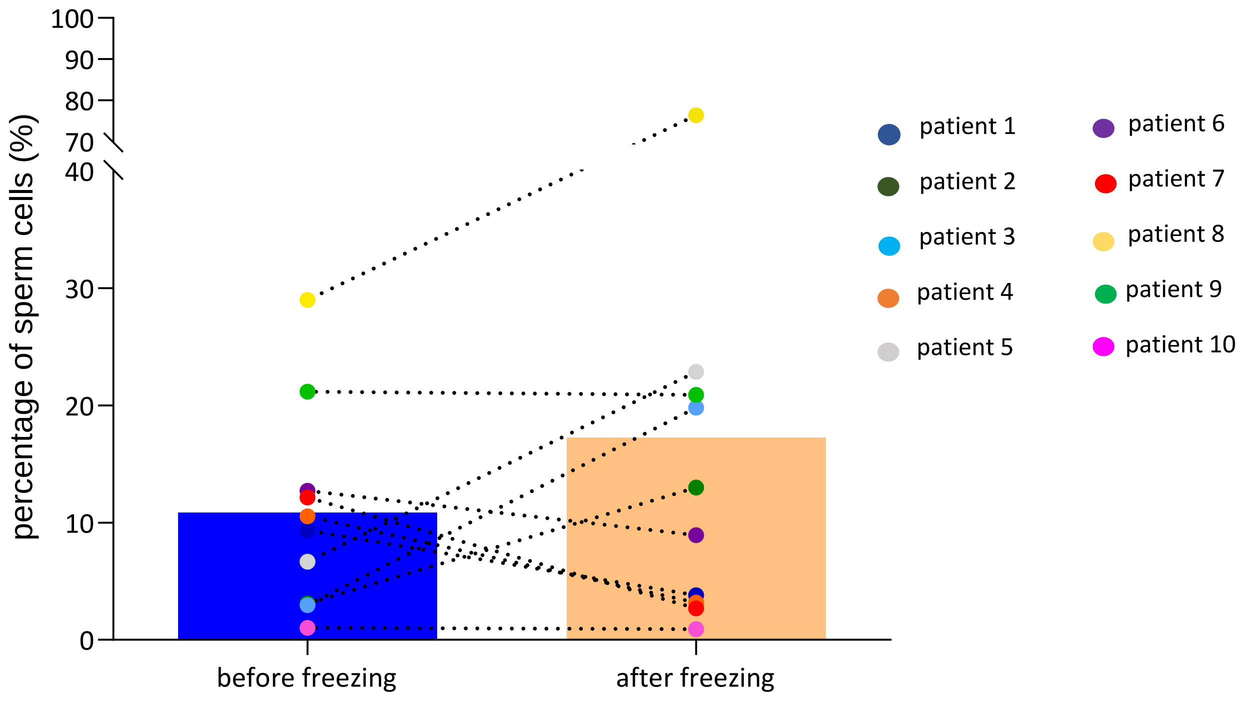

Figure S1: Mean and individual STL distribution of the shortest STL [0; 0.5 a.u.[) measured by Q-FISH before and after freezing-thawing

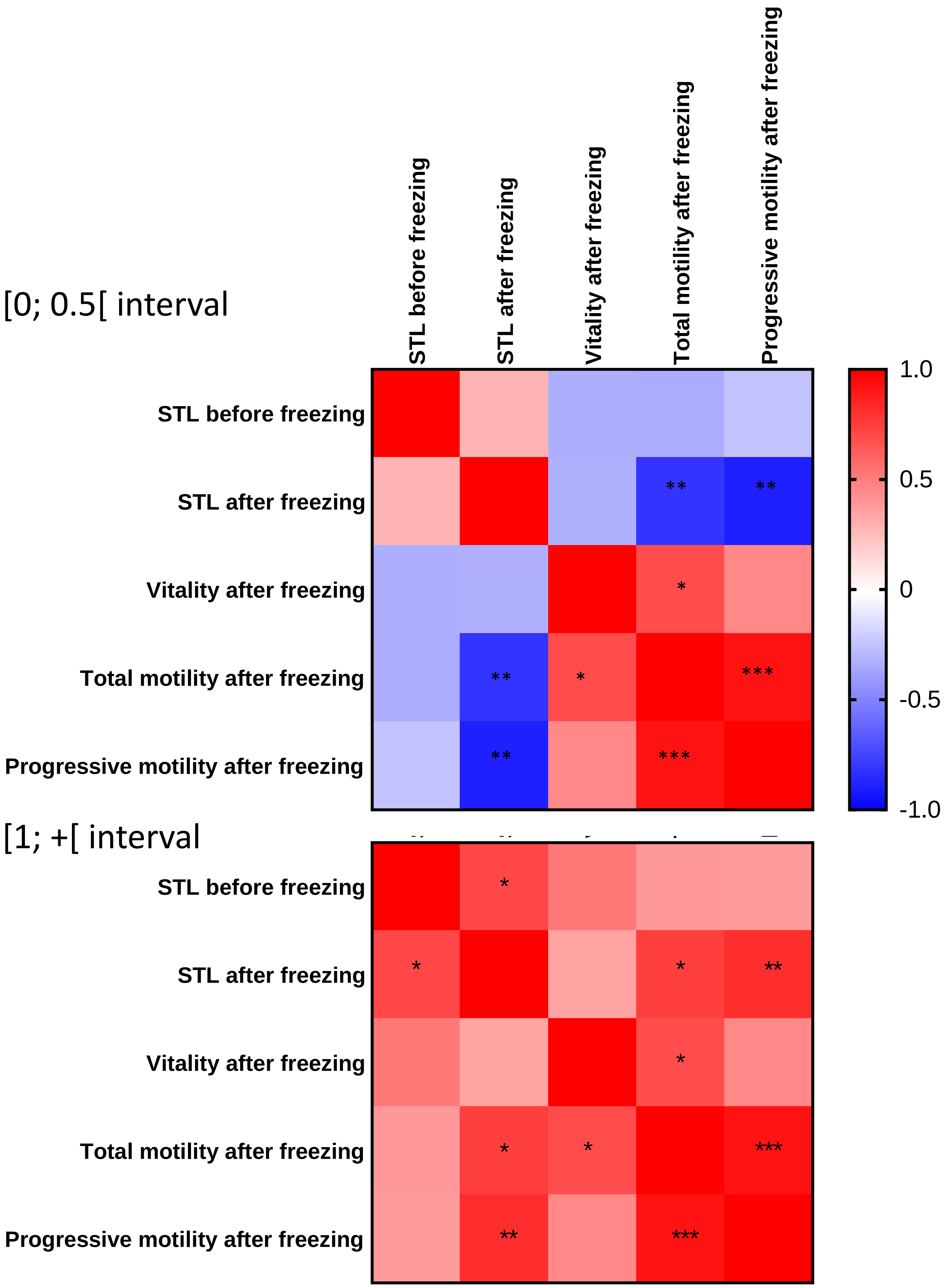

Figure S2: Heatmap of the correlations between the proportion of sperm cells having the shorter [0; 0.5 a.u.[) and the longer STL [1; +[measured by Q-FISH before and after freezing, and sperm parameters for frozen samples
